# An evidence-based health workforce model for primary and community care

**DOI:** 10.1186/1748-5908-6-93

**Published:** 2011-08-06

**Authors:** Leonie Segal, Matthew J Leach

**Affiliations:** 1 Health Economics and Social Policy Group, Sansom Institute, University of South Australia, Adelaide, Australia

## Abstract

**Background:**

The delivery of best practice care can markedly improve clinical outcomes in patients with chronic disease. While the provision of a skilled, multidisciplinary team is pivotal to the delivery of best practice care, the occupational or skill mix required to deliver this care is unclear; it is also uncertain whether such a team would have the capacity to adequately address the complex needs of the clinic population. This is the role of needs-based health workforce planning. The objective of this article is to describe the development of an evidence-informed, needs-based health workforce model to support the delivery of best-practice interdisciplinary chronic disease management in the primary and community care setting using diabetes as a case exemplar.

**Discussion:**

Development of the workforce model was informed by a strategic review of the literature, critical appraisal of clinical practice guidelines, and a consensus elicitation technique using expert multidisciplinary clinical panels. Twenty-four distinct patient attributes that require unique clinical competencies for the management of diabetes in the primary care setting were identified. Patient attributes were grouped into four major themes and developed into a conceptual model: the Workforce Evidence-Based (WEB) planning model. The four levels of the WEB model are (1) promotion, prevention, and screening of the general or high-risk population; (2) type or stage of disease; (3) complications; and (4) threats to self-care capacity. Given the number of potential combinations of attributes, the model can account for literally millions of individual patient types, each with a distinct clinical team need, which can be used to estimate the total health workforce requirement.

**Summary:**

The WEB model was developed in a way that is not only reflective of the diversity in the community and clinic populations but also parsimonious and clear to present and operationalize. A key feature of the model is the classification of subpopulations, which gives attention to the particular care needs of disadvantaged groups by incorporating threats to self-care capacity. The model can be used for clinical, health services, and health workforce planning.

## Background

Disability, morbidity, and mortality associated with chronic disease continue to reflect the dominant source of disease burden in Australia [1] and other high-income countries, and increasingly, in middle-income countries [2]. There is also well-established evidence that the delivery of best-practice care can markedly improve clinical outcomes in patients with chronic disease [3-5] and that best practice involves skilled, multidisciplinary teams [6-9]. But, studies report a discordance between current clinical practice and best-practice guidelines, resulting in poorer outcomes, especially in disadvantaged populations [10,11]. A supportive health infrastructure, adequate health funding and delivery arrangements, and a health workforce matched to healthcare needs will be critical to the delivery of high-quality chronic disease management. The development and distribution of clinical best-practice guidelines is not enough.

### Health workforce planning

The demand (or need) for healthcare gives rise to the demand for the health workforce. Health workforce planning must therefore be underpinned by an understanding of the demand for healthcare. Demand for healthcare can be defined in one of two ways: (1) expressed demand--a market-based concept that reflects purchasing decisions of individuals and insurers--or (2) needs--a more clinically related concept that depends only on the health status of the population and best-practice (cost-effective) care.

Expressed demand will only provide a sound basis for workforce planning where supply and demand meet (or at least approximate) the conditions of the perfect market. But this applies to neither healthcare nor the health workforce [12] because of constraints on supply (*e.g.*, through registration of professions, restrictions on scope of practice and models of care, and limits on education and training places) and demand distorted by pervasive knowledge failures and third-party payment. This means that expressed demand will not reflect informed consumer preferences and, as such, cannot provide a sound basis for health workforce planning. Expressed demand as a basis for health workforce planning is also inconsistent with the adoption of equity as a health system objective.

A needs-based approach to demand is the only valid evidence-based approach to workforce planning. As noted above, needs in this context relate to the concept of clinical best practice, as informed by a combination of efficacy (outcomes in the clinical trial setting), effectiveness (outcomes in the clinic setting), and cost effectiveness (taking into account costs, value for money, and budget constraints).

The challenge is to develop a workforce model that reflects the complexity of the community and clinic population and their needs, is capable of translating those needs into clinical care requirements, and is tractable. This is the challenge addressed by the model described below.

Before moving to that description, we comment briefly on the common use of clinician-population ratios for "workforce planning." Despite the widespread adoption of clinician-population ratios (specified for selected occupations), there is no underlying logic to support their use and no evidence offered to support the selection of a particular ratio as "optimal." The flaws of this approach are well described by Birch and colleagues [13] and other commentators [14].

### Emerging approaches to health workforce planning

There is a small emerging literature on health workforce planning that takes a needs-based approach, attempting to address some of the previously mentioned limitations of health workforce planning. Birch *et al. *[13] have developed an analytical framework for needs-based health human resources planning, which models the impact on the health workforce of various assumptions about the participation rate of providers, their productivity or activity rate, and number of training places [13]. This is a national model that spans across the entire health workforce, which is ideally informed by detailed local data inputs. There is also promising work by Andrews *et al. *[15] with the development of a needs-based, costed, stepped-care model for mental health services. Essentially, their model identifies level of need, the available treatments, and the staff and facilities required to service that need.

While these new approaches to health workforce planning are promising and represent a considerable advance on other approaches, they too are not without limitations. The needs-based component of both of these models essentially assumes the "archetypal" patient, defined solely by their primary medical condition, which as we have argued elsewhere, is insufficient for health workforce planning [16]. We therefore sought to develop a health workforce model that could reflect the diverse nature of the clinic population and their distinct healthcare needs, which not only complements the needs-based approaches to health workforce planning mentioned previously, but challenges these approaches to take a richer perspective on population needs.

### A new approach to health workforce planning

**Figure 1:**
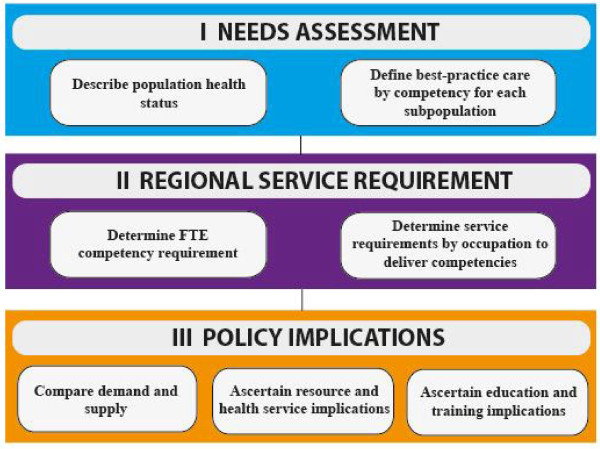
**Needs-based workforce planning framework**. FTE - Full-time equivalent.

Segal *et al. *[17] recently described a needs-based workforce planning framework for estimating the health workforce team (*i.e*., skill mix and hours) required to support the delivery of best-practice care to a regional population. In essence, the framework incorporates three related components that build on each other: (1) a competency- and skill-based needs assessment, (2) an estimate of regional service requirements, and (3) policy implications (see Figure 1). This paper reports on the findings from a recent exercise to operationalize the model through an application to diabetes. The focus here is with the needs assessment, taken to the regional level.

The needs assessment comprises four stages: (1) description of the health status of the population, (2) estimate of the population with defined attributes for the selected region, (3) collation of evidence regarding best-practice care for each identified subpopulation, and (4) translation of best-practice care into care protocols.

### Description of the health status of the population

The first activity was to ascertain patient subpopulations/patient attributes that define the need for a unique clinical team to achieve best-practice diabetes care. This was established through a three-stage process:

• A strategic review of the literature, including a critical appraisal of clinical practice guidelines (CPGs), to identify patient attributes potentially relevant to diabetes management. The search was performed using MEDLINE, EMBASE, and CINAHL, and the following search terms: diabetes mellitus, gestational diabetes, health status, health behavior, patient compliance, self-care, self-efficacy, treatment outcome, type 1 diabetes mellitus, and type 2 diabetes mellitus. The search was limited to papers published in the English language after the year 1990, and for which an abstract was available.

• Discussions with an expert academic panel to identify additional patient attributes. The panel comprised experts in diabetology, diabetes education, community nursing, dietetics, podiatry, cardiology, occupational therapy, and public health; all had clinical expertise in managing diabetes.

• Discussions with cross-disciplinary panels of clinicians working with patients with diabetes in Metropolitan Adelaide (South Australia) or the regional center of Whyalla (South Australia). The aim was to identify additional subpopulations, as well as seek confirmation or adjustment to the key patient attributes identified through the prior processes, using a modified nominal group technique. Essentially, clinicians made comments on the subpopulations in isolation and without influence from the panel. They then shared their suggestions during the panel meetings and discussed the suggestions presented. Findings were iterated to a point of consensus, determined by way of voting. Nineteen clinicians, from 14 disciplines (community nursing, dentistry, diabetes education, dietetics, endocrinology, exercise physiology, general practice, occupational therapy, pharmacy, physiotherapy, podiatry, practice nursing, public health, and social work), were consulted during this process.

**Figure 2:**
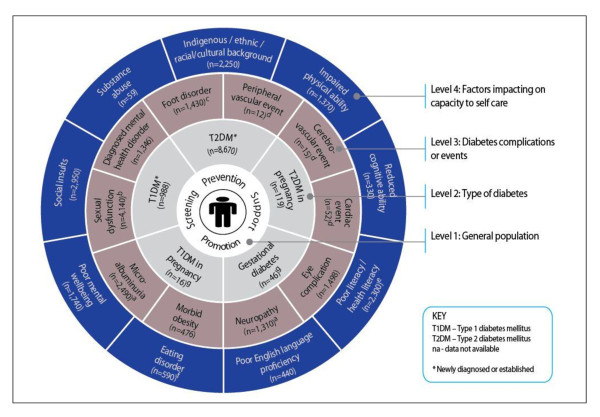
Workforce Evidence-Based (WEB) model for diabetes, with prevalence data*#. *Data represent the number of cases per 10,000 persons with diabetes, which, based on an estimated prevalence rate of known diabetes of 4% [19], equates to a total population of 250,000 persons. #Prevalence data are derived from the Australian Bureau of Statistics National Health Survey (2007-2008) [19], unless specified otherwise. ^a^Australian diabetes, obesity, and lifestyle study (1999/00) [21,22]; ^b^French, Canadian, German, and U.S. surveys of persons aged 16 years and older with any type of diabetes [24-28]; ^c^Amsterdam survey of adults aged 40-94 years with any type of diabetes [29]; ^d^Australian hospital admission data [23]; ^e^U.S. surveys of persons aged 18 years and older with any type of diabetes [30-32]; ^f^German survey of persons aged 18 years and older with any type of diabetes [33]; ^g^Australian Bureau of Statistics birth data (2007) [34].

Twenty-four distinct patient attributes, each requiring a unique occupational and skill mix to manage diabetes in the primary care setting, were identified through this process. Discussions with panel members, and inductive reasoning, led to the clustering of these attributes into four logical and meaningful themes, which were developed into a conceptual model, hereon referred to as the Workforce Evidence-Based (WEB) planning model (see Figure 2).

The four themes/levels of the WEB model are (1) promotion, prevention, and screening of the general or high-risk population; (2) type or stage of disease; (3) complications; and (4) threats to self-care capacity. Level 1 concerns the health workforce to deliver health promotion and primary and secondary prevention services for the "at-risk" population. Levels 2 to 4 concern healthcare needs and the associated health workforce for persons with the diagnosed condition, in our example, diabetes mellitus.

The population with diabetes is characterized by type of diabetes (and whether newly diagnosed) (level 2), whether they have experienced one or more complications or events (level 3), and whether they have attributes that suggest specific threats to self-care capacity (level 4). In this way, a unique set of attributes is attached to each person that would vary over the course of the condition and possibly with life stages. Given the number of potential combinations of attributes (our diabetes model has five level 2 characteristics, ten level 3, and nine level 4), the model can account for literally millions of individual patient types, each with a distinct occupational and skill mix need, with implications for total health workforce required.

### Estimate of the population with defined attributes for selected region

Thirteen databases were identified as potentially suitable for estimating the prevalence of diabetes mellitus and for subpopulations at the national and regional level. No single database contained sufficient information to estimate all 24 subpopulations [18]. The largest, most rigorous, and most accessible data source proved to be the Australian Bureau of Statistics National Health Survey (2007-2008) [19]; this was selected as the primary data source to describe population health status. The National Health Survey had enough detail to generate estimates of specific subpopulations, covering type of diabetes (*e.g.*, type 1, type 2, and gestational diabetes), a range of complications (*e.g.*, morbid obesity, diagnosed mental health disorder, eye disease), functional limitations (*e.g.*, impaired physical ability, reduced cognitive ability), and psychosocial issues (*e.g.*, poor English language proficiency, poor mental well-being, substance abuse, major social/traumatic event, indigenous and ethnic background), based on self-report. This was supplemented by diabetes-specific data sources, such as the Australian diabetes, obesity, and lifestyle study (AusDiab) [20-22], hospital admission data [23], and pertinent survey/other data [24-34]. Prevalence data for diabetes and for each subpopulation, based on a typical area/local health service population of 250,000 persons, are reported in Figure 2, drawing on a combination of Australian and international data.

### Collation of evidence regarding best-practice care for each identified subpopulation

The original plan was to describe best-practice care for each subpopulation from CPGs. However, this process proved problematic. While we identified 27 published diabetes CPGs that looked suitable for our purpose, none of the guidelines adequately captured all 24 patient subpopulations, with most ignoring threats to self-care capacity (level 4 of the WEB model); even with the combination of guidelines, gaps still remained. The collective guidelines of the Canadian Diabetes Association, American Diabetes Association, and the National Collaborating Centre for Chronic Conditions provided adequate coverage for many subpopulations [16]. For the patient attributes for which CPGs offered limited or no guidance, a consensus of expert opinion was used, employing the cross-disciplinary panels of clinicians and the modified nominal group technique described earlier.

### Translation of best-practice care into care protocols

**Figure 3:**
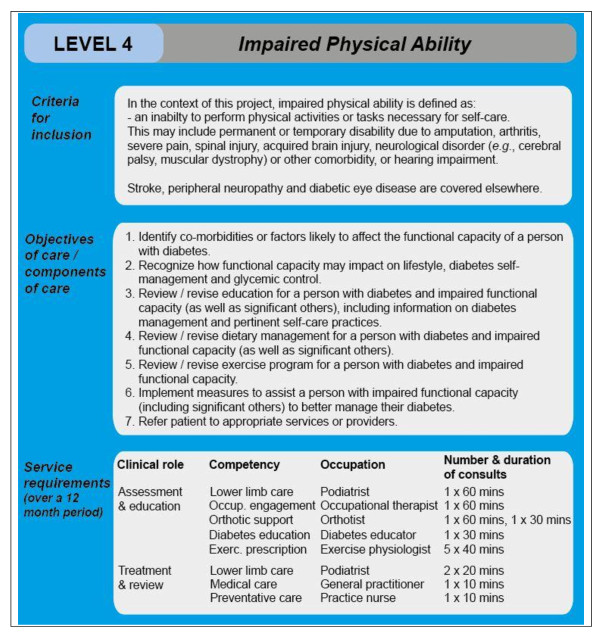
**Example of a clinical care protocol--the "impaired physical ability" module of the WEB model**.

Descriptions of best practice (largely defined by objectives of care) were translated into clinical care protocols (Figure 3)--expressed as the number and duration of consults by competency and skill level. Initially, it was expected that this information could be extracted from CPGs. However, the guidelines rarely contained adequate details to allow the required clinical input to be ascertained [16]. A decision was therefore made to again use the collective experiences, opinions, and knowledge of the cross-disciplinary panels of clinicians to assist with the translation of best-practice care into clinical care protocols and to reach consensus on the protocols through an iterative process using a modified nominal group technique (as previously described). The clinical protocol in effect sits behind each attribute/module.

The care protocols, together with the population estimates for each patient attribute, are then fed into phase two of the workforce model to estimate the regional demand for each competency (*i.e*., hours × competency/person/year). This is then mapped onto possible occupations, with alternative ways of delivering defined competencies modeled, for example, to reflect a predominant specialist or generalist approach to service delivery. More intricate mapping, which takes into consideration the inherent complexity of translating competencies into care protocols and occupations (*i.e*., taking into account variations in occupational mix and productivity, role substitution, and the diversity of staff attributes, such as level of experience, competency), was beyond the scope of this project. Further work in this area would be valuable. The estimation of regional demand is the intended next step of the project, with findings expected to be published soon.

## Discussion

The WEB model, which emerged as an essential component of the needs analysis, presents a new and effective way of approaching health workforce planning. The modular structure allows different components of care to be easily added or omitted according to the characteristics or attributes of the population, best-practice care, evidence of cost effectiveness, and changes in the understanding of disease and threats to successful treatment. Also, because the care protocols within each module/subpopulation are competency-based, it can easily accommodate emerging service providers and the modelling of alternative delivery methods.

The WEB model has clear implications for the desirable composition of the multidisciplinary team. In particular, occupations that are trained to deliver competencies pertinent to level 4 (threats to self-care), such as social work, occupational therapy, or mental health workers, are more likely to be identified as core members of the primary care team. An evidence-based approach to establishing the desirable mix of the multidisciplinary team, and using that for service planning, is fundamental to the delivery of high-quality care in which knowledge can be translated into clinical practice for the benefit of the patient.

An unexpected benefit of the WEB model is its diversity of application. While the model was primarily designed to guide health workforce planning, health services planning, and funding that reflects care needs, it also provides a useful framework to direct group discussion and workshops around chronic disease management and to identify pertinent gaps in data and research. For instance, it has already highlighted important limitations in the production of CPGs and the evidence base on which they are drawn [16]. The WEB model may also benefit clinicians by providing a new way of thinking about the delivery of individualized clinical care and provide a framework for health screening.

There are several challenges to implementation of the WEB model. One challenge relates to the quality of data inputs. Determining the level of service need in complex patients is also problematic. Even where it is possible to define with strong agreement the management requirements for each subpopulation, questions still remain about how to combine workforce and service needs across attributes, within and between levels. The expectation is that a simple additive approach will be applicable across levels, as each level deals with quite distinct types of needs. However, within a level, recognizing that clinicians can cover more than one issue during a consultation (where these are related), the protocol is to adopt the conservative position of taking the highest value (of consultation time) for the competency at that level (and not add across attributes). The result will be a minimum estimate of workforce and service need. Combining up to an entire primary and community care chronic disease team, which is the ultimate aim, adds an extra level of complexity, reflecting the genuine challenges of this research/policy question.

We are confident that the workforce planning framework and the WEB model can be usefully applied to other groups of conditions, such as cardiovascular disease, mental health disorders, or musculoskeletal disorders, recognizing that some modification to the structure, levels, and attributes may be required. Discussions with health workforce planning agencies have confirmed the value of the model for those seeking an evidence-based approach to health workforce and health services planning. The South Australian Department of Health is already using the WEB model to inform the planning of state-wide pediatric speech pathology services and the state's palliative care workforce.

## Summary

This paper discusses our learnings from the operationalization of a needs-based workforce planning framework. The WEB model, a major output of this work, offers a means for incorporating the diversity of the clinic population while retaining simplicity of design. This work represents a critical step in the development of a workforce model that reflects the complexity of the community population and their needs.

The composition of the multidisciplinary team that arises out of application of this model is very different to that which emerges under a more narrow medical condition focus; for example, social work is identified as a core competency in the primary care team. By incorporating threats to self-care into the model, and into health workforce and health services planning, the model offers the promise of a service system better able to serve the needs of groups who are disadvantaged, who undoubtedly are disadvantaged under planning models that focus on the archetypal, generally high-functioning patient. The ultimate promise is better health outcomes for all, including a reduction in avoidable health deficits for persons with multiple disadvantages.

## Competing interests

The authors declare that they have no competing interests.

## Authors' contributions

LS and MJL contributed equally to the writing of the manuscript, from conception to submission. Both authors read and approved the final manuscript.
